# Childhood undernutrition mediates the relationship between open defecation with anemia among Ethiopian children: a nationally representative cross-sectional study

**DOI:** 10.1186/s12889-024-18931-x

**Published:** 2024-06-03

**Authors:** Biniyam Sahiledengle, Pammla Petrucka, Fikreab Desta, Yordanos Sintayehu, Telila Mesfin, Lillian Mwanri

**Affiliations:** 1 Department of Public Health, Madda Walabu University Goba Referral Hospital, Bale-Goba, Ethiopia; 2 College of Nursing, University of Saskatchewan, Saskatoon, Canada; 3 Department of Medicine, Madda Walabu University Goba Referral Hospital, Bale-Goba, Ethiopia; 4 Equity and Human Flourishing, Research Centre for Public Health Research, Torrens University Australia, Adelaide Campus, Adelaide, SA 5000 Australia

**Keywords:** Anemia, Stunting, Wasting, Underweight, Open defecation, Children, Mediation analysis

## Abstract

**Background:**

Poor sanitation and/or open defecation are a significant public health problem in Ethiopia, where access to improved sanitation facilities is still limited. There is a growing body of literature about the effect of open defecation on children’s linear growth failure. However, very few studies about the effects of open defecation on child anemia exist. In this study, we examine whether childhood undernutrition (i.e. stunting, wasting, and underweight) mediates the relationship between open defecation and childhood anemia in children aged 6–59 months in Ethiopia.

**Methods:**

We used pooled Ethiopia Demographic and Health Survey data (2005–2016) comprising 21,918 (weighted data) children aged 6–59 months. Anemia was defined as an altitude-adjusted hemoglobin (Hb) level of less than 11 g/deciliter (g/dl) for children under 5 years. Childhood undernutrition was assessed using height-for-age Z-scores (HAZ), weight-for-age Z-scores (WAZ), and weight-for-height Z-scores (WHZ) for stunting, wasting, and underweight respectively. Mediation effects were calculated using the bootstrap and the indirect effect was considered significant when the 95% bootstrap confidence intervals (95% CI) did not contain zero. Moreover, separate multilevel regression analyses were used to explore the statistical association between open defecation and child anemia, after adjusting for potential confounders.

**Results:**

Our analysis revealed that nearly half (49.6%) of children aged 6 to 59 months were anemic, 46.8% were stunted, 9.9% were wasted, and 29.5% were underweight. Additionally, 45.1% of children belonged to households that practiced open defecation (OD). Open defecation was associated with anemia (AOR: 1.28; 95% CI: 1.18–1.39) and it positively predicted anemia with direct effect of β = 0.233, *p *< 0.001. Childhood undernutrition showed a partial mediating role in the relationship between OD and anemia. Analyzing the indirect effects, results revealed that child undernutrition significantly mediated the relationship between open defecation and anemia (stunting (βindirect = 0.014, *p* < 0.001), wasting (βindirect = 0.009, *p* = 0.002), and underweight (βindirect = 0.012, *p* < 0.001)). When the mediating role of child undernutrition was accounted for, open defecation had a positive impact on anemia with a total effect of βtotal = 0.285, *p* < 0.001.

**Conclusion:**

Open defecation showed a significant direct effect on anemia. Child undernutrition remarkably mediated the relationship between OD and anemia that further magnified the effect. This finding has an important programmatic implication calling for strengthened, accelerated and large-scale implementation of strategies to end open defecation and achieve universal access to sanitation in Ethiopia.

## Introduction

According to the World Health Organization (WHO), open defecation (OD) is defined as “the practice of defecating in open areas, such as in the fields, forests, or in the bodies of water, rather than in a designated toilet or latrine” [1, 2]. In low-middle income countries (LMICs), poor sanitation or open defecation is a significant public health and environmental concern. The burden of OD at the global level is substantial, with an estimated 673 million people practicing OD worldwide in 2019, and over 1.5 billion people lacking basic sanitation services, such as private toilets or latrines [1, 3]. Of those who still practice open defecation, 90% of people reside in three regions including sub-Saharan Africa (SSA) [4]. On the recognition of the health impact of open defecation in populations, one of the aims of the United Nations' Sustainable Development Goals (SDGs) is to end open defecation by 2030, as indicated in SDG target 6.2 [5]. Overall, in the last few decades, open defecation has significantly decreased globally; however, significant regional disparities exist, with SSA uniquely experiencing a high rate of OD [6].

Defecating in the open is an affront to dignity and is one of the risk factors for children's poor health outcomes. The effect of open defecation on child health is substantial and multi-faceted, and because of their immature immune systems, children are affected by a host of diarrheal illnesses [7, 8]. The conditions that are most commonly associated with OD are small intestine bacterial overgrowth, soil-transmitted helminthiases, environmental enteropathy, stunting, and an increased burden of anemia [9]. In addition to the direct effects on child health, OD also has indirect consequences, contributing to environmental pollution through contaminating agricultural land, food sources, and drinking water [10, 11]. This scenario can impact food security, facilitate the spread of food and waterborne diseases, and exacerbate undernutrition in communities where open defecation is prevalent [9, 10].

A growing number of studies have shown that open defecation continues to compromise children’s growth [12–16], and is associated with an increased risk of stunting [8, 14, 17–19], wasting [8, 20], and underweight [19, 21]. For instance, an ecological analysis in India showed that a 10 percent increase in open defecation was associated with a 0.7 percentage point increase in both stunting and severe stunting [19]. Another cross-sectional study reported that compared with open defecation, household access to a latrine facility was associated with 16–39% reduced odds of stunting in children [22]. However, the relationship between open defecation and child anemia has received much less attention [23, 24].

A few studies have attempted to identify the effect of OD on child hemoglobin levels and anemia in LMICs [13, 24]. Anemia, more specifically iron deficiency, usually manifests after six months of age and may worsen if iron-rich supplemental foods are not consumed in appropriate amounts. At least 50% of cases of anemia are caused by iron deficiency; however, this percentage can vary depending on the location [25]. There are two plausible pathways that open defecation can result in childhood anemia including: related to intestinal parasites such as parasitic and geohelminth infections, specifically ascariasis, hookworm [26–28], schistosomiasis [29, 30]; and via a condition known as environmental enteropathy, spread of infectious diseases. The common hypothesis suggests that when child playgrounds become heavily contaminated with fecal matter, children are exposed to fecal bacteria and pathogens regularly, establishing a cycle wherein recurrent infections occur. This perpetual cycle of recurrent infections can lead to nutritional deficiencies and anemia in children [9]. In the case of extended infections, the ability of young children to absorb essential nutrients may become compromised, which can also lead to anemia. Consequently, anemia can contribute to increased mortality, impaired physical development, poor cognitive abilities, and impaired immune response in young children [31–34].

In Ethiopia, OD remains the most serious public health problem, notably in rural settings, where 37.7% of the population are open defecators [35]. Reportedly, although between 2005 and 2016 Ethiopia experienced a notable decrease in the prevalence of open defecation [36], globally, this nation still ranks among the top countries that practice OD, holding the third position after Nigeria and Indonesia [37]. In the same way, the prevalence of anemia and childhood undernourishment remains endemic in Ethiopia affecting 57% and 38% of under-five children, respectively [38]. These interconnected scourges have devastating consequences on public health [39, 40].

The Government of Ethiopia has undertaken significant efforts to enhance the health and well-being of children by focusing on improving their nutritional status and ensuring universal access to water, sanitation, and hygiene (WASH) services [41, 42]. This commitment is exemplified through the development of key initiatives such as the One WASH program [43] and other flagship programs to 'End Open Defecation' [44]. Additionally, Ethiopia has been implementing different strategies to ensure food and nutrition security, such as the National Nutrition Programs I and II (2008–2020) and the Seqota Declaration Roadmap (2015–2030) [41, 42]. The WASH efforts have shown promising results with increased sanitation coverage and improved child growth, but considerable efforts are still required to significantly reduce OD in rural and urban areas in Ethiopia [36, 38].

There is a growing recognition of the interconnectedness between various childhood health indicators, including undernutrition and anemia. Exploring whether childhood undernutrition acts as a mediator in the relationship between OD and childhood anemia is essential for understanding the underlying mechanisms and pathways through which OD may affect anemia prevalence. In this regard, mediation analysis provides valuable insights into understanding complex causal pathways and mechanisms underlying relationships between these variables. To the best of our knowledge, there has been no prior population-level study that has assessed the association of OD with anemia and examined childhood undernutrition (i.e. stunting, wasting, and underweight) as a potential mechanism. Previous studies investigated different forms of child undernutrition [45–52] and anemia [53–57] in Ethiopia, however, information regarding the association of OD with anemia is limited. Given the limited evidence on the relationship between OD and child anemia, the objective of this study was twofold: [1] to investigate the association between OD and anemia in children 6–59 months of age in Ethiopia, and [2] to examine whether childhood undernutrition (i.e. stunting, wasting, and underweight) mediates the relationship between open defecation and childhood anemia in children aged 6–59 months in Ethiopia. Our study contributes to the ongoing nutrition-sensitive efforts, including improving access to sanitation to improve child nutrition and contributing to Sustainable Development Goals (SDG) goals 2 and 6. Additionally, this study contributes to the currently scant literature available on the mediating role of child undernourishment in OD and anemia relationships.

## Methods

### Conceptual model

**Fig. 1:**
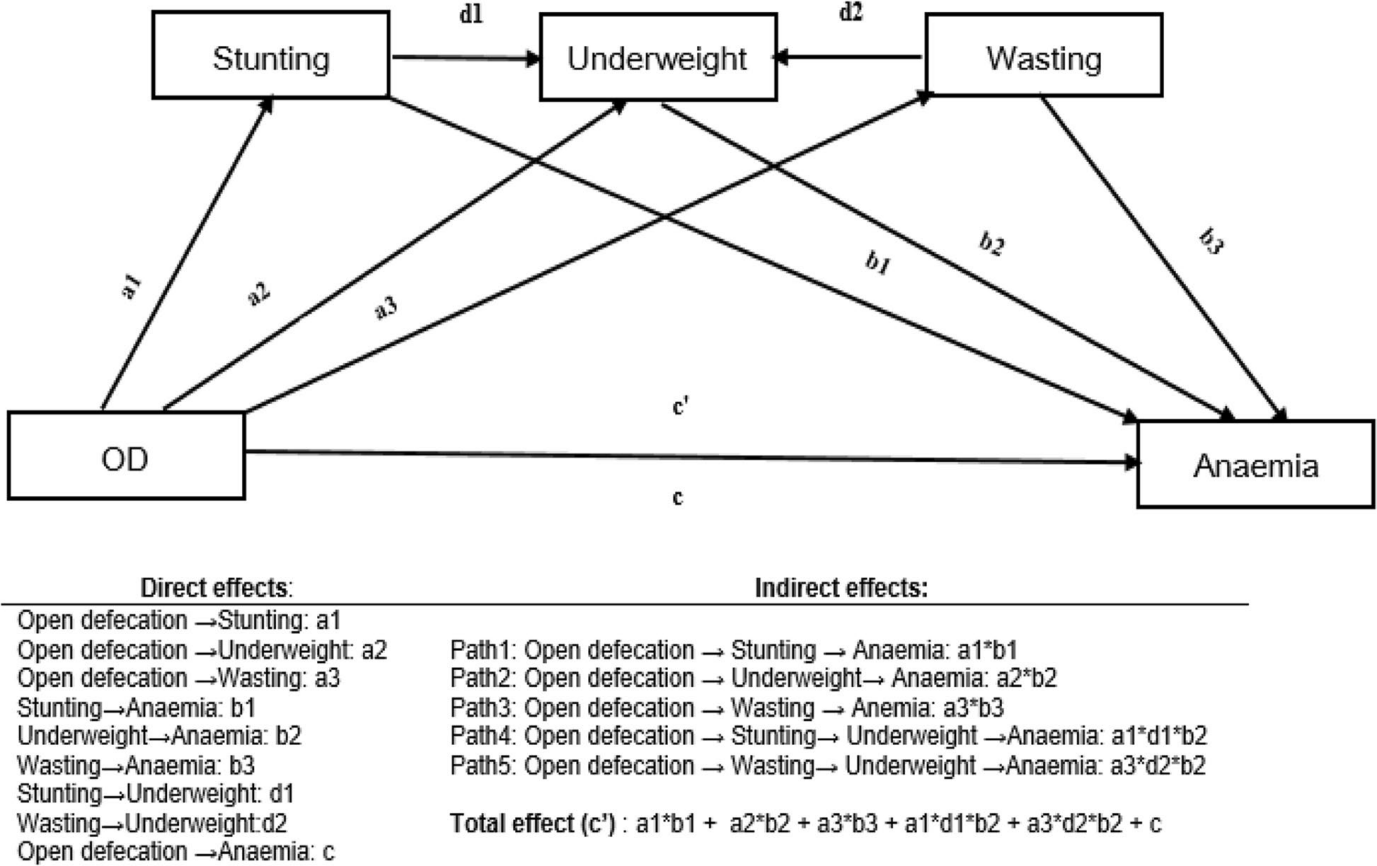
Conceptual model of the association between open defecation and child anemia

The study’s hypothetical parallel and serial mediation analysis conceptual model is presented in Fig. 1. Mediation analysis is a statistical method used to test and quantify the extent to which the mediator variable accounts for the relationship between the independent and dependent variables [58]. In mediation analysis, the relationship between an independent variable (X) and a dependent variable (Y) is examined to determine if this relationship is partially or fully mediated by a third variable known as the mediator (M). It involves estimating the direct effect of the independent variable on the dependent variable, the indirect effect mediated through the mediator, and the total effect (sum of direct and indirect effects) [59, 60]. The mediator variable helps to explain the relationship between the independent and dependent variables by identifying the intermediate steps or mechanisms through which the effect occurs. In this study, the main mediator variables were those related to childhood undernutrition (i.e., stunting, wasting, and underweight).

The effect of open defecation on child health and undernutrition is substantial and multi-faceted. We used mediation analysis to understand the underlying process or mechanism by which an independent variable (OD) affects a dependent variable (anemia). This approach helps in determining whether the relationship between the dependent and independent variables is direct or if it operates through an intervening variable. The most commonly known cause of anemia in children is iron deficiency anemia, which is closely linked to open defecation [61, 62]. Based on the available literature we hypothesized positive direct effects between open defecation and child anemia. Recent evidence from analysis of 47 demographic and health surveys found that in 65% of countries children exposed to an open sanitation facility had higher odds of being anemic [24]. Studies evidenced that the risk of soil-transmitted helminths (STHs) increases with the practice of open defecation [63, 64], contributing to the high burden of anemia in children. Furthermore, open defecation has a direct impact on child undernutrition. There was strong literature support on the effects of open defecation and child undernutrition [8, 14]. In areas where open defecation was common, there was a high burden of infections, which can lead to nutrient deficiencies and malabsorption, causing stunted growth in children [65, 66]. Evidence showed that OD is estimated to account for 54% of the variation in average child height in LMICs [67].

Nutritional deficiency, such as stunting in children, is also associated with a high burden of anemia. A systematic review and meta-analysis found that under-five anemia was positively associated with child undernourishment [68, 69]. Undernourished children are more susceptible to infections and illnesses, which can lead to anemia due to increased demand for iron and other nutrients to support the immune system. We hypothesized positive direct effects between childhood undernutrition and anemia, concerning the three mechanisms as presented in Fig. 1. We also hypothesized that each of these mechanisms might act as a mediator in relation to the effect of OD with anemia.

Stunting and wasting are two forms of undernutrition that can lead to underweight in children through complex and interconnected relationships. Stunting primarily affects a child’s height, it can also contribute to reduced body weight as a result of poor nutrition and repeated infection. Chronic malnutrition can lead to reduced muscle mass and subcutaneous fat, contributing to underweight in children. Wasting, on the other hand, often caused by acute food shortages and/or disease, directly impacts a child’s weight-for-height ratio. When a child is wasted, they have a significantly low body weight for their height, which can lead to being classified as underweight. An underweight child may be stunted, wasted, or both [70, 71]. In this regard, mediation analysis provides valuable insights into understanding complex causal pathways and mechanisms underlying relationships between these variables.

If a mediation effect is to be established, then it should fulfill the following criteria: [1] the independent variable (open defecation) should have a strong influence on the dependent variable (child anemia); (2) the independent variable (open defecation) should be strongly related to mediators (stunting, wasting and underweight); (3) the independent variable (open defecation) and mediators should be related to the outcome variable. However, if the open defecation is no longer significant when the mediator variables are controlled, the finding will be a full mediation effect. If the independent variable still shows a significant association when the mediator is controlled, the finding can be considered as a partial mediation effect [72].

### Data source and study design

Data from the Ethiopian Demographic and Health Survey (EDHS) from 2005, 2011, and 2016 rounds were analyzed. The EDHS is a cross-sectional, nationally representative survey. EDHS employs a two-stage multistage, sampling weights, and cluster sampling method to establish a representative sample of households at the national and regional levels [35, 39, 73]. The EDHS collected blood samples among all children aged 6 to 59 months included in the survey for hemoglobin tests using a battery-operated portable HemoCue analyzer (*HemoCue®*) (www.hemocue.com). Weight was measured with an electronic mother-infant scale (SECA 878 flat) designed for mobile use. Height was measured with a measuring board (Shorr Board®). Children younger than age 24 months were measured lying down on the board (recumbent length) while standing height was measured for the older children. Further information regarding the survey methodologies and measurement of nutritional status is presented in the full EDHS reports [35, 39, 73].

### Outcome variable

The study’s dependent variable was childhood anemia. Anemia was described based on the WHO cutoff point, and a hemoglobin level of less than 11 g/deciliter (g/dl) for children aged 6–59 months old was categorized as anemic. The EDHS has computed hemoglobin by adjusting for different altitude levels, since a lower effective hemoglobin count as altitude increases [74, 75].

### Exposure variables

The main exposure variable was open defecation (yes, no). Households that lack sanitation facilities or defecate on bush or field/forest/bodies of water, were considered as open defecators [36, 76].

### Mediators

The main mediator variables were childhood undernutrition (i.e., stunting, wasting, and underweight which calculates in three nutritional indices: height-for-age, weight-for-height, and weight-for-age, respectively). Stunting was defined as height-for-age Z-scores (HAZ) below minus two standard deviations (-2SD) from the median of the reference population. Wasted was defined as children whose weight-for-height Z-scores (WHZ) are below minus two standard deviations (-2SD) from the median of the reference population, and are too thin for their height. Children whose weight-for-age Z-scores (WAZ) measures below minus two standard deviations (-2SD) from the median of the reference population are underweight for their age. All anthropometric variables were constructed based on the 2006 World Health Organization (WHO) child growth standards [77].

### Control variables

A control variable, also known as a covariate, is a variable that is included in an analysis to account for potential confounding factors or to control for their influence on the relationship between the independent and dependent variables. The control variables used in this study were generated based on literature [8, 51, 53, 56, 69, 78, 79] and their availability in the EDHS dataset. The sex of the child (male, female), age of the child (6–11 months, 12–23, 24–35, and 36–59 months), perceived size of the child at birth (large, average and small), received deworming medication in the last 6 month (yes, no), received iron supplementation in the last 6 months (yes, no), mother's age (< 18, 18–24, 25–34, or >  = 35), mother's education (no education, primary and above), mother's current work status (yes, no), household wealth index (poor, middle, and rich), place of residence (rural, urban), and region. We used the wealth index constructed by the DHS; as a proxy measure derived from asset ownership. The wealth index was calculated using the principal components analysis (PCA) method to represent the household wealth index as a score of household assets. The wealth index was categorized into five quintiles (e.g., poorest, poorer, middle, richer, richest).

### Data analysis

Descriptive statistics were used to summarize participant characteristics. The *'Svy'* commands were employed to allow for adjustments for the cluster-sampling design and weight as recommended by DHS. Details about the sampling weighting procedure can be found in the EDHS report [35, 39, 73]. We used Pearson’s chi-squared tests to assess differences in child anemia frequencies by respondents’ characteristics. We applied a multilevel logistic regression model to investigate the associations of household OD practices with child anemia among children aged 6 to 59 months [53, 80]. We first estimated the unadjusted association that did not adjust for any control variables, and then estimated the adjusted association from models that adjusted for all control variables. We have taken into account, various factors when selecting variables for inclusion in a multivariable regression model. These factors include the theoretical basis of the variable, its practical significance, and its statistical significance in bivariate analysis (with a *p*-value < 0.20). Given the hierarchical structure of the EDHS data, where children are nested within households and households within clusters, we employed a multilevel mixed-effects model. This model incorporated both fixed and random effects to appropriately account for the nested nature of the data. Accordingly, we ran five models adjusted for control variables, mediators, and a combination of both. In all models, OD practices showed a significant association with anemia at a *p*-value of < 0.05 in the multilevel multivariable logistic regression analysis. For that reason, the association between our main exposure variable (OD) and anemia was further examined for the mediation effect of the potential mediators.

A mediation analysis was used to test whether stunting, wasting, and underweight could mediate the relationship between OD (independent variable) and child anemia (dependent variable). Mediation analysis was used to investigate the mechanism through which an independent variable influences a dependent variable by introducing a mediator variable. There are different types of mediation, including complete mediation and partial mediation (i.e. independent variable has both direct and indirect effects on a dependent variable). Mediators can also be classified as single and sequential [59, 60]. Serial mediation hypothesizes a causal chain linking the mediators (stunting, wasting, and underweight), with a specified direction flow (For instance: OD → stunting → underweight → anemia).

Generalized Structural Equation Modeling (GSEM) was used to test the mediation effect of the potential parallel and sequential mediators on child anemia. Given that our outcome variable was binary, it was analyzed assuming a Bernoulli response distribution and a logit link function. The mediation analysis was performed using Stata ‘*gsem’* and the ‘*nlcom’* command to estimate the direct, indirect, and total effects of OD on child anemia. Bootstrapping with 5000 samples was used, and potential control variables were controlled for in the models. Mediation is significant if the 95% bias-corrected confidence intervals (CIs) for the indirect effect does not include zero [58, 81]. We reported adjusted odds ratios (AORs) along with 95% confidence intervals (CIs) at a significance level of p < 0.05 in our multivariable multilevel analysis to represent the strength and direction of the association between OD and anemia. In our analysis, multicollinearity is not considered a problem if the variance inflation factor (VIF) values are less than five [82]. All analyses used STATA/MP version 14.1 (Stata Corp, College Station, TX, USA).

## Results

### Characteristics of the study population

Table 1 shows the participants’ characteristics. In this analysis, a total of 21,918 (weighted data) children aged 6–59 months with complete hemoglobin records were included in this study. The median age of the study participants was 30 months (Interquartile range (IQR): 14–45 months) and 51.2% were male. A total of 45.1% of children were from households that practiced open defecation. A total of 70.5% of mothers had no education, 45.6% of the participants were from poor households, and 89.4% were from rural settings. In our analysis, 49.6% (95%CI: 48.9–50.3) of children aged 6–59 months suffered from anemia. Our analysis also revealed that 46.8% of children aged 6–59 were stunted, 9.9% were wasted, and 29.5% were underweight.

**Table 1:** The weighted distribution of socio‐demographic characteristics of the sample population and prevalence of anemia among children 6–59 months by characteristics of the study population, 2005-2016 (*n* = 21,918,EDHS-2005, *n* = 4,259; EDHS-2011, *n* = 9,259; EDHS-2016, *n* = 8,399)

**Variables**	**Weighted n (%)**	**Children with anemia, n (%)**	*p*-value^a^
***Outcome***			
* Prevalence of anemia (95%CI)*		*49.6 (48.9–50.3)*	
***Exposure variable***			
** Open defecation**			*P* < 0.001
Yes	9,404 (45.1)	4,942 (47.7)	
No	11,467 (54.9)	5,409 (52.3)	
***Mediators***			
***Child undernutrition status***			
** Stunting**			*p* < 0.001
Yes	9,885 (46.8)	5,158 (49.2)	
No	11,241 (53.2)	5,319 (50.8)	
** Wasting**			*p* < 0.001
Yes	2,094 (9.9)	1,283 (12.2)	
No	19,032 (90.1)	9,194 (87.8)	
** Underweight**			*p* < 0.001
Yes	6,231 (29.5)	3,470 (33.1)	
No	14,895 (70.5)	7,007 (66.9)	
***Control variables***			
** Sex**			0.051
Male	11,220 (51.2)	5,547 (51.3)	
Female	10,698 (48.8)	5,267 (48.7)	
** Age (months)**			*p* < 0.001
6–11	2,481 (11.3)	1,676 (15.5)	
12–23	4,716 (21.6)	2,961 (27.4)	
24–35	4,629 (21.2)	2,308 (21.4)	
36–59	10,028 (45.9)	3,851 (35.7)	
** Birth interval**			*p* < 0.001
< 33 months	15,046 (68.6)	7,594 (70.2)	
≥ 33 months	6,872 (31.4)	3,219 (29.8)	
** Size of child at birth**			*p* < 0.001
Larger	7,029 (32.2)	3,532 (32.7)	
Average	8,867 (40.6)	4,229 (39.2)	
Small	5,950 (27.2)	3,025 (28.1)	
** Currently breastfeeding**			*p* < 0.001
Yes	15,491 (70.7)	8,102 (74.9)	
No	6,427 (29.3)	2,712 (25.1)	
** Diarrhea**			*p* < 0.001
Yes	3,199 (14.6)	1,776 (16.4)	
No	18,688 (85.4)	9,028 (83.6)	
** Received deworming medication in that last 6 month**			*p* < 0.001
Yes	3,037 (13.8)	1,303 (12.1)	
No	18,881 (86.1)	9,510 (87.9)	
** Iron supplementation**			0.199
Yes	1,356 (6.2)	652 (6.0)	
No	20,562 (93.8)	10,161 (94.0)	
** Vitamin A last 6 months**			*p* < 0.001
Yes	10,834 (50.4)	5,106 (48.1)	
No	10,681 (49.6)	5,509 (51.9)	
** Mother's age**			*p* < 0.001
< 18	106 (0.5)	56 (0.5)	
18–24	4,668 (21.3)	2,452 (21.3)	
25–34	11,536 (52.6)	5,684 (52.6)	
≥ 35	5,609 (25.6)	2,621 (25.6)	
** Mother's education**			*p* < 0.001
No education	15,457 (70.5)	7,730 (71.5)	
Primary and above	6,461 (29.5)	3,083 (28.5)	
** Mother's currently working**			*p* < 0.001
Yes	6,674 (30.5)	3,137 (29.0)	
No	15,238 (69.5)	7,675 (71.0)	
** Wealth index**			*p* < 0.001
Poor	10,002 (45.6)	5,352 (49.5)	
Middle	4,624 (21.1)	2,224 (20.6)	
Rich	7,292 (33.3)	3,237 (29.9)	
** Residence**			*p* < 0.001
Urban	2,326	915 (8.5)	
Rural	19,592	9,898 (91.5)	
** Region**			*p* < 0.001
Agrarian	11,947	5,209 (48.1)	
Pastoralist	9,456	5,392 (49.9)	
City administration	515	213 (1.9)	
** EDHS**			*p* < 0.001
2005	4,260	2,029 (18.8)	
2011	9,259	3,964 (36.7)	
2016	8,398	4,820 (44.5)	

^a^Pearson chi^2^

### The association between open defecation and anemia

After conditioning on the potential control variables, significant associations between OD and anemia were found (AOR: 1.28; 95% CI: 1.18–1.39). Anemia and OD were found to be strongly associated, even after controlling for possible mediators both individually and collectively (Table 2).

**Table 2:** Multilevel logistic regression analysis on the association between open defecation and anemia among children aged 6–59 months in Ethiopia, EDHS (2005–2016)

Variable	Child anemia					
	**Crude OR, 95%CI**	**A**	**B**	**C**	**D**	**E**
		**Adjusted OR, 95%CI**	**Adjusted OR, 95%CI**	**Adjusted OR,95%CI**	**Adjusted OR, 95%CI**	**Adjusted OR, 95%CI**
**Open defecation**						
Yes	1.44 (1.36–1.53)**	1.27 (1.17–1.37)**	1.29 (1.19–1.39)**	1.28 (1.18–1.38)**	1.27 (1.17–1.38)**	1.28 (1.18–1.39)**
No	Ref.	Ref.	Ref.	Ref.	Ref.	Ref.

A: Model adjusted for sex, age, birth interval, perceived size at birth, received deworming medication in the last six months, received iron supplementation in the last six months, mother's age, mother's education, mother's occupation, wealth index, residency, region and survey year (lists of potential covariates included in the subsequent models)

B: Model adjusted for all potential covariates + Stunting

C: Model adjusted for all potential covariates + Wasting

D: Model adjusted for all potential covariates + Underweight

E: Model adjusted for all potential covariates + Stunting + Wasting + Underweight

^**^p < 0.001; The model accounted for clustering and representativeness

### Mediation analysis

**Fig. 2:**
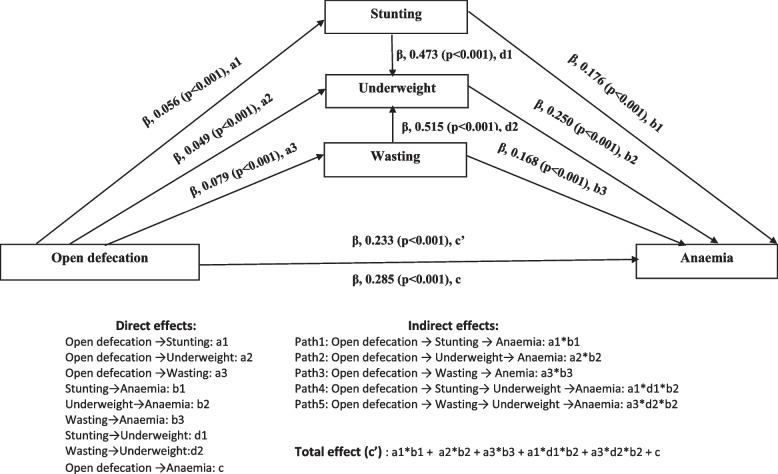
Multiple mediation model for the direct and indirect effects of open defecation on child anemia. The three mediators (i.e. childhood stunting, wasting, and underweight) were simultaneously entered. The bootstrapping method with bias-corrected confidence estimates (based on 5,000 bootstrap samples) was used to test our mediational hypotheses. Significant at the 0.05 level

Table 3 illustrates findings from the mediation analysis. Figure 2 presents the relationship between the independent variables and mediators as well as the mediators and the outcome variables, which were all significant. The result revealed that open defecation positively predicts child anemia (β_direct_ = 0.233, *p* < 0.001). With the inclusion of the mediating variables (stunting, wasting, and underweight) the effect of open defecation on anemia was slightly increased and remained statistically significant (β_total_ = 0.285, *p* < 0.001).

**Table 3:** Bootstrapping direct, indirect, and total effects and 95% confidence intervals (CI) for the mediational analysis in the relationship between open defecation and anemia among children aged 6–59 months in Ethiopia, EDHS (2005–2016)

Effect	Path	β^a^ coefficient (effect)	Bootstrap Std.err	Bootstrap 95%CI		*p*-value	Proportion of total effect that is mediated
				**LLCI**	**ULCI**		
Direct	Open defecation → anemia	0.233	0.035	0.164	0.302	*p* < 0.001	
Indirect 1	Open defecation → stunting → anemia	0.014	0.003	0.008	0.020	*p* < 0.001	4.9
Indirect 2	Open defecation → wasting → anemia	0.009	0.003	0.003	0.015	0.002	3.2
Indirect 3	Open defecation → underweight → anemia	0.012	0.002	0.007	0.017	*p* < 0.001	4.2
Indirect 4	Open defecation → stunting → underweight → anemia	0.009	0.002	0.006	0.013	*p* < 0.001	3.2
Indirect 5	Open defecation → wasting → underweight → anemia	0.007	0.001	0.004	0.009	*p* < 0.001	2.5
Total	Total indirect effect	0.052	0.005	0.042	0.062	*p* < 0.001	18.2
	Total effect (Open defecation → anemia)	0.285	0.035	0.216	0.355	*p* < 0.001	

^a^The models were adjusted for sex, age, birth interval, perceived size at birth, received deworming medication in the last six months, received iron supplementation in the last six months, mother's age, mother's education, mother's occupation, wealth index, residency, region and survey year; Bias-corrected bootstrapped CIs, 5000 bootstrap samples; LL- Lower Limit, UL- Upper Limit

The result also revealed a significant indirect effect of open defecation on anemia through childhood stunting (β_indirect_ = 0.014, *p* < 0.001), wasting (β_indirect_ = 0.009, *p* = 0.002), and underweight (β_indirect_ = 0.012, *p* < 0.001). This implies the relationship between open defecation and anemia is partially mediated by childhood undernutrition. The total indirect effect of open defecation on anemia was also significant (β_total indirect_ = 0.052, *p* < 0.001).

This study also revealed that the association between OD and anemia was partially mediated by the sequential mediators (stunting/wasting and underweight). After conditioning on childhood stunting and underweight, the effect of open defecation on anemia was significant (β_indirect_ = 0.009, *p* < 0.001). Similarly, after conditioning on wasting and underweight, open defecation remains a significant predictor of anemia (β_indirect_ = 0.007, *p* < 0.001).

Stunting, wasting, and underweight accounts for 4.9%, 3.2%, and 4.2% of total effect. The sequential mediators, childhood stunting and underweight, mediated 3.2% of the total effect of open defecation on anemia. Likewise, wasting and underweight, mediated 2.5% of the total effect of open defecation on anemia. The proportion of the total indirect effect that was mediated was 18.2% (Table 3).

## Discussion

The act of defecating in the open exposes children to unhygienic conditions, making them susceptible to ingesting harmful pathogens, such as parasites and bacteria that can lead to various health complications, including anemia. We hypothesized that the practice of OD will positively predict child anemia. Additionally, it was hypothesized that child undernutrition mediated this relationship. To our knowledge, this is the first study to investigate the relationships and underlying pathways between OD and anemia among children aged 6–59 months in Ethiopia. The study showed significant associations between OD and child anemia. The results of the mediation analyses showed that OD was related to anemia both directly and indirectly through child undernutrition.

Our analyses suggest that the prevalence of anemia remains high (49.6%) among children aged 6–59 months in Ethiopia. The high burden of anemia in the country was higher than the World Health Organization (WHO) threshold (i.e. above 40% anemia prevalence) classified as a major public health issue [83]. Current projections towards SDG 2030 suggest that it is unlikely for Ethiopia, as well as for many low-income countries and nations, to completely eradicate child anemia by 2030 [84]. Ethiopia's slow progress toward eliminating child anemia has implications for calling for urgent action and understanding of the reasons why eliminating child anemia remains an uphill battle for the country. For instance, evidence indicated that the prevalence of anemia in Ethiopian children increased significantly between 2011 and 2016, rising from 44 to 57% which might need an in-depth-investigation of what was behind [57].

On the other hand, anemia prevalence rates reported in the Ethiopian National Micronutrient Survey (2016) was 34.4% [85]. In addition, the baseline survey of the National Food and Nutrition Strategy (2023) reported the prevalence of anemia among 6–59 months old children to be 16% [86]. These surveys offer valuable insights into the evolving landscape of the prevalence of anemia in Ethiopia. The Micronutrient Survey and the National Food and Nutrition Strategy survey classify the magnitude of anemia in Ethiopia as moderate and the latter as mild, respectively. This is the lowest prevalence of childhood anemia reported so far by national surveys in Ethiopia, contrasting with the EDHS-2016 national surveys, which reported a higher prevalence of anemia (57%) [38]. This underscores the importance of continued surveillance and research efforts to accurately assess the prevalence of anemia among young children in this nation. We allude that the observed discrepancy in the prevalence of anemia among these surveys could probably be attributed to factors related to the lab tests and procedures applied or any other factor.

The high burden of OD in Ethiopia might have contributed to the existing prevalence of anemia in the country. Open defecation can lead to contamination of the environment, which increases the risk of feco-orally transmitted diseases caused by intestinal helminths, protozoans, bacteria and viruses [87]. Intestinal parasites consume nutrients from the intestines and blood stream and disrupt nutrient absorption leading to micronutrient deficiencies, including iron, vitamin B12, protein, and others ultimately causing anemia [58, 88–91]. Bacterial and viral entero-pathogens change the morphology of intestinal villi, promote systemic inflammatory response, increase gut permeability causing reduced nutrient absorption and loss of nutrients [92, 93]. There will also be increased demand for nutrients due to immune response to the infection that exacerbates the situation leading to anemia. Studies reported that OD is associated with low hemoglobin concentrations and a higher prevalence of anemia in children [94].

There is compelling evidence that nutritional factors do not account for more than two-fifths of cases of anemia. Micronutrient interventions are likely to have limited impact, and current interventions should be broadened to include ending OD as well as effective anti-hookworm measures [28]. Therefore, it is essential to enact extensive measures to combat the concerning high burden of anemia in Ethiopian children, by not only enhancing their nutritional status but also bolstering nutrition-sensitive initiatives such as providing access to WASH facilities. By addressing the issue of OD and improving access to WASH facilities, we can contribute to reducing the prevalence of anemia among children in Ethiopia and ultimately promote better overall child health and development.

The trend of OD in Ethiopia has decreased considerably from 61.9% in 2005 to 32.3%% in 2016, an average decline of more than 3 percentage point per year [35, 39]. The latest EMDHS survey conducted in 2019 reported a further decline in OD (27.1%) [40]. The trend reflects a significant improvement in sanitation coverage and progress toward achieving universal access to safe sanitation facilities in the country. Despite the promising notable decrease in OD over the years, still a significant number of the Ethiopian population practiced OD.

Our study showed that more than two-fifths (45.1%) of children were from households that practiced OD, which is similar to that obtained from the studies from in low-income settings such as Ghana, 49.8% [95] and India, 54%[96]. On the other hand, our finding was much higher than the pooled estimates of OD practice in SSA, 22.5% [6], and the Joint Monitoring Program (JMP) of WHO and UNICEF 2021 report, 18% [97]. These findings highlight the persistent issue of OD in low-income settings and its potential impact on public health, including the increased risk of anemia in children. Therefore, addressing OD practices and improving sanitation can play a crucial role in reducing the occurrence of anemia and iron deficiency.

The total effect is an aggregate of direct and indirect effects. In this study, the positive total estimated effect indicates a direct relationship between OD and anemia. The direct associations we observed in this study suggest that there is strong evidence to support that open defecation correlated with a higher prevalence of anemia, through contamination of environmental surroundings. OD exposes children to fecal pathogens and increases the risk of STHs, infections, and diarrheal disease, all of which may have a cyclic effect on anemia. When children suffer from diarrhea, for instance, they lose essential nutrients, which are necessary for proper growth and development. This reduction of essential nutrients and absorption contributes to the development of micronutrient deficiencies, these can exacerbate and perpetuate the occurrence of anemia in children [68, 69]. In alignment with our results, a recent meta-analysis by Larsen et al. found that living in communities with poor sanitation facilities was associated with higher odds of anemia [13]. Existing literature reported similar findings regarding the effect of poor sanitation on anemia prevalence [24, 78, 98].

The proposed hypotheses in this study were verified in the results of the mediation analysis. The current study illustrated that childhood undernutrition mediated the association between OD and anemia. Iron deficiency and iron deficiency anemia are correlated with childhood malnutrition [99]. Our study finding suggests that OD is a significant contributor to stunting, wasting, and underweight because it increases the risk of diarrheal and other diseases that can cause malabsorption of nutrients, which, in turn, can increase the risk of anemia in children [61].

Childhood undernutrition mediates the association between open defecation and anemia by exacerbating the negative effects of poor sanitation and hygiene practices. Undernourished children are more susceptible to infections and illnesses, which can lead to anemia due to increased demand for iron and other nutrients to support the immune system. Undernutrition weakens the immune system, making children more susceptible to infections and illnesses caused by exposure to fecal pathogens from OD, contributing to anemia through reduced absorption of nutrients [61, 75, 99, 100]. Ultimately, undernutrition amplifies the detrimental effects of open defecation on the risk of anemia by lowering the body's resilience and ability to combat infections and childhood illnesses. Infections can contribute to anemia through various pathways that include reduced intake and absorption of nutrients, increased loss of nutrients, increased demand for nutrients, impaired hemoglobin formation and red blood cell production [101, 102]. Overall, there exist four potential pathways by which undernutrition in children results in anemia. Initially, by means of inadequate consumption of nutrients. Children who are malnourished may not be receiving enough vital nutrients from their food, such as iron, vitamin B12, and folate, which increases the risk of anemia. Secondly, by decreasing the formation of red blood cells, malnutrition in children can result in an impairment in the body's ability to produce red blood cells, which may play a role in the onset of anemia. Thirdly, children who are malnourished are more prone to infections and illnesses. These infections and illnesses can cause anemia because the body needs more iron and other nutrients to strengthen the immune system. Undernourished children may experience poorer intestinal absorption of nutrients, which can result in iron, vitamin B12, and folate deficiencies-all of which are critical for preventing anemia [61, 89, 99, 101].

### Strength and limitation of the study

One of the limitations of the mediation analysis is the potential for unmeasured confounding, which occurs when there are unobserved variables that influence both the mediator and the outcome variable. Another limitation involves the study design, where the cross-sectional or observational data cannot establish causal relationships between variables, and the prevalence of anemia and undernutrition and its direct and indirect factors over time would not have been taken into consideration. The unavailability of longitudinal data limits the support of a definite model in our study. Moreover, our findings are limited to children aged 6–59 months and, as such, any interpretation of our findings with regards to other demographics and populations must be undertaken with caution. We also acknowledge that the mediation analysis may not have captured all possible pathways or mechanisms through which the independent variable influences the outcome variable. Finally, in our model, some biological and behavioral characteristics that may affect anemia were not considered. Despite these limitations, the significance of our study and its findings are emphasized by the utilization of a nationally representative sample and rigorous analysis techniques. Moreover, the findings contribute evidence concerning the mediating role in the relationship between OD and anemia.

## Conclusion

Our study demonstrated that there was a statistically significant association between OD and anemia. It also revealed that childhood undernutrition has a partial mediating role in the relationship between OD and anemia. These findings have an important programmatic implication, calling for strengthened, accelerated, and large-scale implementation of strategies to end open defecation, achieve universal access to sanitation, and thereby attain Sustainable Development Goal 6 in Ethiopia. It is recommended that Ethiopia step up implementation of the existing initiatives like One WaSH, Co-WASH, and The Global WASH for eradication of the practice of open defecation. While mediation analysis can provide insights into potential causal pathways, it cannot confirm causality without experimental or longitudinal evidence. Hence, a prospective longitudinal study is required to better estimate the causal effect of OD and its associated mediators on child anemia in low-income settings.

## Data Availability

No datasets were generated or analysed during the current study.
